# Lithography Assisted Fiber-Drawing Nanomanufacturing

**DOI:** 10.1038/srep35409

**Published:** 2016-10-14

**Authors:** Behrad Gholipour, Paul Bastock, Long Cui, Christopher Craig, Khouler Khan, Daniel W. Hewak, Cesare Soci

**Affiliations:** 1 Centre for Disruptive Photonic Technologies, Nanyang Technological University, 637371, Singapore; 2 Optoelectronics Research Centre, University of Southampton, Southampton SO17 1BJ, UK

## Abstract

We present a high-throughput and scalable technique for the production of metal nanowires embedded in glass fibres by taking advantage of thin film properties and patterning techniques commonly used in planar microfabrication. This hybrid process enables the fabrication of single nanowires and nanowire arrays encased in a preform material within a single fibre draw, providing an alternative to costly and time-consuming iterative fibre drawing. This method allows the combination of materials with different thermal properties to create functional optoelectronic nanostructures. As a proof of principle of the potential of this technique, centimetre long gold nanowires (bulk T_m_ = 1064 °C) embedded in silicate glass fibres (T_g_ = 567 °C) were drawn in a single step with high aspect ratios (>10^4^); such nanowires can be released from the glass matrix and show relatively high electrical conductivity. Overall, this fabrication method could enable mass manufacturing of metallic nanowires for plasmonics and nonlinear optics applications, as well as the integration of functional multimaterial structures for completely fiberised optoelectronic devices.

Optical fibre technology has revolutionized many aspects of modern life. Since the late 60’s, optical fibre applications have widened from conventional optical telecommunications to high power lasers and sensors^1^,^2^. Typical optical fibres for telecommunication are drawn from glass preforms. Within the fibre drawing process, the preform is fed into the furnace at a specified speed and softened above the glass transition temperature to be subsequently pulled into a fibre. The fibre necks down within the furnace with a given diameter determined by the draw speed^3^,^4^. Within this process, the aspect ratio of the preform features are maintained, enabling the creation of complex photonic structures like microstructured glass fibres, as well as conventional core/clad structures^5^.

In recent years, this process has evolved into a multimaterial realm, where families of fibres composed of low melting point (T_m_ and T_g_ < 500 °C) conductors, semiconductors and insulators have been demonstrated for a wide variety of applications including, metamaterials^6^,^7^,^8^, light sources^9^, photodetectors^10^, sensors^11^,^12^, medical imaging^13^ as well as microsphere production^14^,^15^.

Such fibres could be realized in a range of functional materials including amorphous, crystalline, semiconducting and metallic compounds^16^,^17^,^18^,^19^,^20^,^21^,^22^,^23^,^24^ using a limited number of methods: “stack and draw” of polymer fibres for low melting point materials, vapor deposition into microstructured glass preforms for crystalline semiconductors, as well as the Taylor-wire and melt-filling methods for metallic cored glass fibres.

The polymer stack and draw technique is based on the encapsulation of bulk functional cores in preforms, using iterative size reduction techniques in conjunction with rolled polymer films to achieve multimaterial components within a fibre platform across a range of sizes^25^,^26^,^27^. So far, this was the only method allowing size reduction of the active cores down to the nanometre scale, limiting the use of multimaterial fibre manufacturing for nanophotonic applications.

A number of techniques have been demonstrated to specifically incorporate metal cores into glass fibres, most notably the Taylor-wire method^28^,^29^,^30^ that involves using a metal wire held in glass tubes (typically borosilicate). One end of the tube is closed and subsequently heated in order to soften the glass to a temperature at which the metal is in liquid state and the glass can be drawn. This allows the production of a fine glass capillary containing a metal core. Alternatively, metallic cores have also been obtained within fibres of different geometries by pressure-assisted melt-filling of drawn microstructured fibres^31^,^32^,^33^,^34^.

## Litbography assisted fiber drawing (LAFD)

Here we demonstrate a flexible method, called lithography assisted fibre drawing (LAFD), for producing functional nanowires and nanowire arrays enclosed in a glass fibre with a single fibre draw. This method employs planar fabrication techniques, and relies on thin films for the realization of nanowires or nanoscale components of different materials, with vastly different bulk thermal properties. As a proof of principle, we applied this method to demonstrate metal-cored silicate fibres with continuous and electrically conductive Au nanowires with radii down to 150 nm.

**Figure 1:**
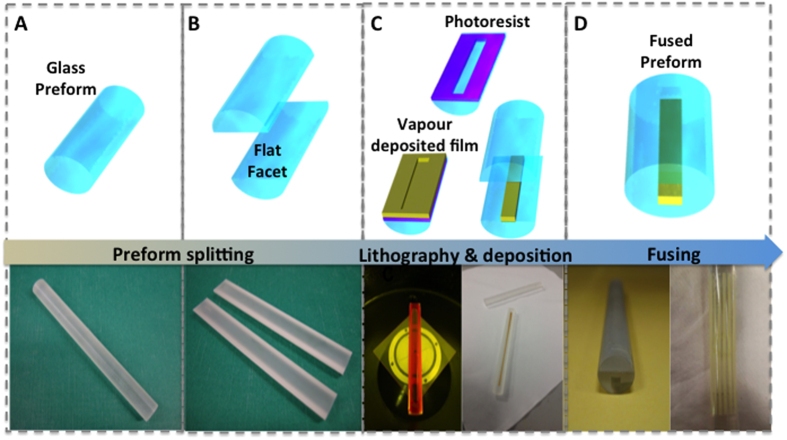
Planar fabrication assisted fibre nanomanufacturing. A glass preform (**A**) is cut to reveal two flat facets after polishing (**B**). Photolithography is used to pattern the facet and conventional planar deposition techniques along with the lift-off process allows microscale patterns of, in-principle, any metal/semiconductor to be deposited. Corresponding photo shows split and polished preform with photoresist (left) and single gold line deposited using e-beam evaporation technique (right) (**C**). A final fusing procedure allows the realisation of a planar assisted fibre preform for nanowire manufacturing (**D**).

The concept behind LAFD is to embed a thin film of the material to be incorporated within the fibre, through the bulk glass preform. This has a two-fold advantage: first, conventional planar lithographic methods can be used to pattern thin films with desired geometries to be scaled down through the drawing process, and second the melting temperature of thin films can be matched to that of glass by controlling deposition conditions and exploiting substrate effects to induce melting point depression^35^,^36^,^37^,^38^. The overall process involves three main steps for the preparation of the multimaterial fibre preform. As shown in Fig. 1, these include: i. glass preparation, in which the glass preform is cut into parts and the facets are polished; ii. functional core preparation, in which planar lithography (photolithography, stencil lithography, etc.) and vapour deposition are used to add functional films and define the desired patterns on the facets; and iii. preform fusing, in which the preform slices are put back together to reconstruct the original shape of the preform. Subsequently, conventional single step fibre drawing is carried out.

In this demonstration, commercial silicate glass rods (Schott glass N-SF8), 10 mm in diameter and 10 cm long, are used as the preform material (Fig. 1A). The glass rod is cut in half along the length to create two flat facets. Then, lapping, polishing and cleaning of the cut facets are followed in sequence to yield a flat and optically smooth surface for planar fabrication (Fig. 1B). This also aids the homogenous spinning of photoresist and better surface adhesion of deposited layers. One of the polished facets is used to spin coat the photoresist required to define micron scale patterns by photolithography. To realise embedded nanowires with a single fibre draw, we defined stripes along the preform with typical widths of 1000 μm (Fig. 1C).

It has been established that the physical properties of nanoparticles, nanowires and thin films produced by conventional planar deposition techniques are different to those of the same bulk materials. Such thin films tend to conform to a degree to the properties of their substrates and their respective attributes are greatly influenced by the deposition conditions and dimensions^39^. In particular, their melting point is depressed compared to their bulk counterparts, particularly for metals^40^,^41^,^42^,^43^,^44^. According to the Gibbs-Thompson theory, due to the presence of nanometre-size grains, vapour deposited films can melt hundreds of degrees below the corresponding bulk forms. For nanoparticles with large surface-to-volume ratio, the binding energy of surface atoms is greatly reduced, resulting in lower thermal energy required to free the atoms from the solid, hence a lower melting temperature. A phenomenological description of this effect can be derived from classical thermodynamics and is summarised by the Gibbs-Thompson equation:


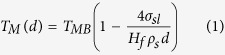


where *T*_*M*_ is the nanoparticle melting temperature, *T*_*MB*_ the bulk melting temperature, *H*_*f*_ the bulk heat of fusion, *σ*_*sl*_ the solid-liquid interface energy, *ρ*_*s*_ the density of the solid phase, and *d* the nanoparticle diameter. Accordingly, a gold thin film with grains of the order of *d* = 5 nm would melt at a temperature as low as 839 °C (for bulk Au, *T*_*MB*_ = 1064 °C, *H*_*f*_ = 67 kJ.Kg^−1^, *σ*_*sl*_ = 0.27 J.m^−2^, and *ρ*_*s*_ = 19.3 × 10^3^ g.cm^−3^)^45^,^46^.

**Figure 2:**
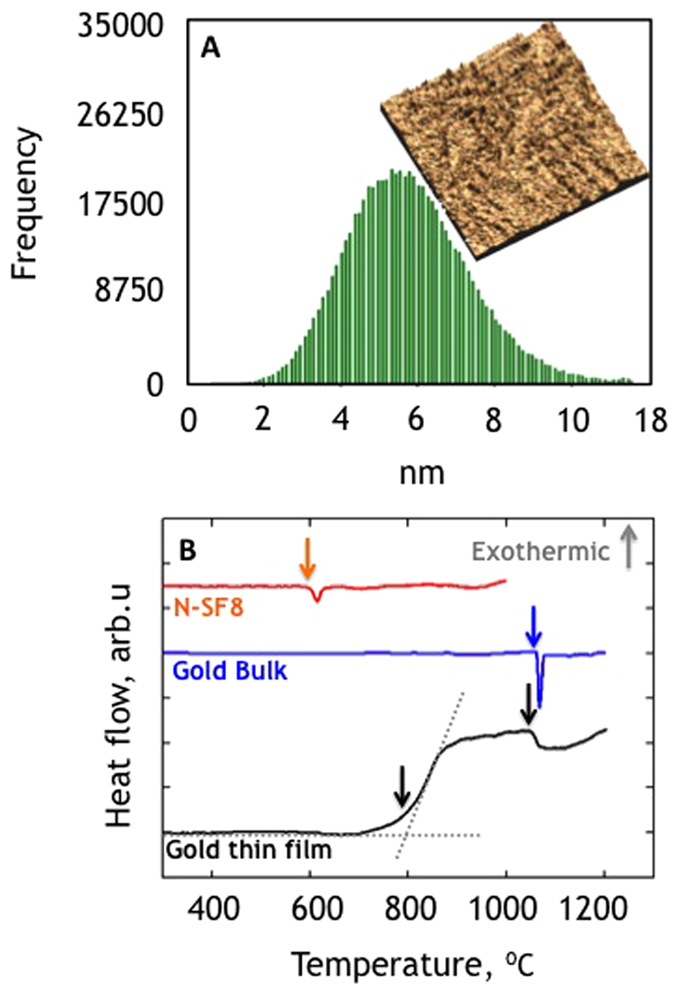
Metallic thin films on polished preforms. (**A**) Roughness analysis of Au film on the preform showing an average roughness of 5.6 nm. inset) Corresponding atomic force microscopy topographic image of Au thin film deposited on a polished glass perform. Image shows a 2 μm × 2 μm scan. (**C**) Thermogravimetric/differential thermal analysis (Tg/DTA) of gold thin film (thickness = 100 nm, in black), bulk gold (blue) and N-SF8 Schott preform glass.

In our experiments, we found that the evaporated Au films have a surface roughness of the order of 6 nm, as determined by AFM surface topography (Fig. 2). This is consistent with thermogravimetric/differential thermal analysis (Tg/DTA) in Fig. 2B, showing the contrast in thermal properties of bulk and thin film Au in comparison to that of N-SF8 preform glass. N-SF8 and bulk Au have thermogravimetric signatures at 1064 °C and in the region of 800 °C, which are assigned to their respective glass transition and melting points (indicated by arrows). For the measurements of the thin film, Au is deposited conformally onto the inner wall of a Tg/DTA crucible. In this case, the thermogravimetric curve shows an upward trend starting from ~700 °C, which stabilizes at ~850 °C, followed by a second dip at ~1064 °C. We interpret this behaviour as arising from the flow of the molten film along the crucible walls beginning at 700 °C, upon which molten liquid Au gathers in a pool at the bottom of the crucible and solidifies until reaching the bulk melting point at 1064 °C (see Experimental Section). The intercept between the baseline and the linear portion of the thermogravimetric curve (dotted lines) can be taken as the melting point of the Au thin film (T_m_ ≈ 800 °C). The depression of thin film melting point shown here demonstrates the potential of our technique in enabling the drawing of fibres with embedded nanoscale features of materials with vastly different thermal properties to the preform, by way of controlling the film deposition conditions as opposed to utilising bulk constituents.

Following lithographic patterning of the design, planar thin film deposition techniques such as e-beam, thermal evaporation, sputtering or low temperature CVD techniques can be utilised to deposit the desired material with the desired properties, on the surface of the patterned preform, followed by “lift-off” or etching of the resist. Subsequently, the two halves of the preform are fused back together in a vacuum furnace (Fig. 1D). For highly reactive functional materials, which would oxidize on exposure to the atmosphere, an optional capping layer made from the preform material could be deposited to prevent oxidation or contamination after deposition, and to improve adhesion during the fusing process. Finally, a single step fibre draw is carried out to scale the preform and obtain an optical fibre embedded with nanostructured components. The drawing temperature for these planar assisted preforms must be set above the softening point of the preform material (for N-SF8 glass, T_10_^7.6^ = 678 °C) and the melting point of the Au thin film.

It should be noted that by adjusting the temperature and choosing a glass clad with appropriate T_g_, one can tune the viscosity of the glass clad with respect to the depressed melting point of the gold core. At high drawing temperatures, the glass softens and the core collapses, losing its predefined rectangular structure and converting into a cylindrical shape, thus resulting in embedded metal nanowire cores. At lower drawing temperature, one may be able to preserve the predefined rectangular structure of the core within the glass clad.

### Ultra-Long Nanowires

**Figure 3:**
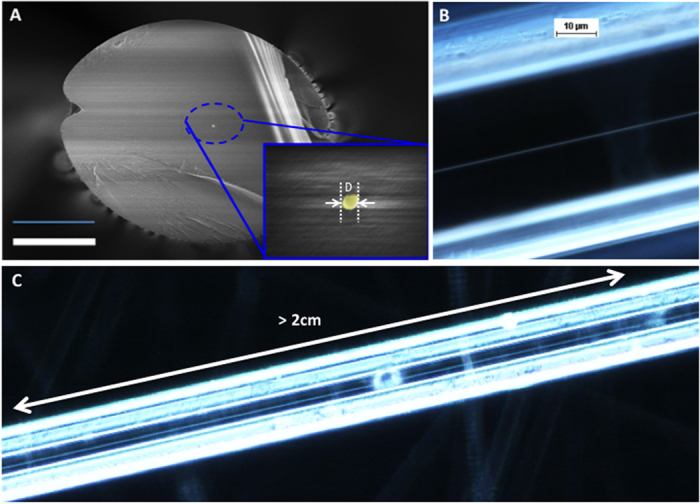
Au nanowires embedded in glass fibre. (**A**) Scanning electron microscopy image of single nanowire of D = 330 nm diameter enclosed in a silicate glass fibre. Scale bar = 30 μm. (**B**) Dark field microscopy side view of a drawn fibre, revealing the nanowire core. (**C**) Dark field microscopy of ultra-long piece of fibre with continuous core (>2 cm).

Ultra-long Au gold nanowires embedded in silicate glass clads were produced by LAFD starting from a preform patterned with 1 μm thick and 1000 μm wide lines, at a drawing temperature of T = 850 °C (Fig. 3). The diameter of the embedded nanowires is governed by the dimensions of the original planar features deposited on the facet of the preform and the drawing parameters. As in conventional fibre drawing, the fibre clad diameter (scaling ratio) is controlled by the feed rate and the drum/capstan speed. Since LAFD preserves the aspect ratio of the preform features, a patterned core line thickness of 1 μm, width of 1000 μm (cross sectional area of 1000 μm^2^), and a circular preform diameter of ∅ = 10 mm drawn down to ∅' = 100 μm (aspect ratio of ~1 × 10^−5^) are expected to yield a circular cross section nanowire with diameter of D~315 nm. This is in excellent agreement with the actual 330 nm diameter measured in the case of Fig. 3A.

**Figure 4:**
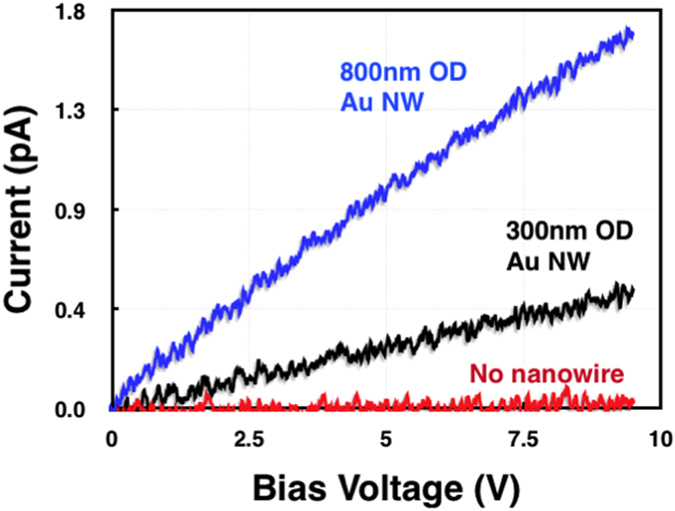
Conductivity of ultra-long nanowires. Current-voltage characteristics of 3 cm long gold nanowires with diameters of 800 nm (blue) and 300 nm (black), compared to an empty glass fibre control.

Electrical integrity of the long Au nanowires was tested by conductivity measurements along macroscopic distances (~3 cm), using silver conductive paint at the fibre tips to make contact to the nanowire core (Fig. 4). The measured current-voltage characteristics show a clear Ohmic behaviour with the expected dependence on nanowire widths, and a clear contrast with a control fibre of the same diameter without the nanowire inclusion. The resistance of the Au nanowires is 5.5 × 10^12^ and 5.5 × 10^13^ Ω for the 800 and 300 nm diameters, respectively.

Indeed the resistance of the nanowire can be calculated using the Pouillets Law:


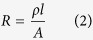


where R is resistance, *ρ* is bulk resistivity, l is length of the nanowire and A is the cross sectional area. Given the bulk resistivity value of Au, length and diameter being 2.44 × 10^−8^ Ω·m, 3 cm, and 800 nm, respectively, the resistance is calculated as 1.46 × 10^3^ Ω. However, nanowire conductivities are highly dependent on their fabrication technique, interfaces and lattice arrangement. Furthermore, an increase in resistance with increasing length is expected and widely observed, and in this case, variations in thickness can also occur across 3 cm length. Therefore, throughout literature, electrical resistance measurements in metallic and semiconductor nanowires typically show higher values than those calculated based on a simple bulk resistivity formula and a wide-ranging set of resistances have been reported^47^,^48^,^49^. Note also that the actual nanowire conductivity may be reduced as a result of high contact resistance resulting from less than ideal contact to the core tips, as well as the presence of nano-cracks within the fibre, as no particular care was taken while handling the fibres to prevent bending (this may be alleviated by the development of post-draw annealing processes).

**Figure 5:**
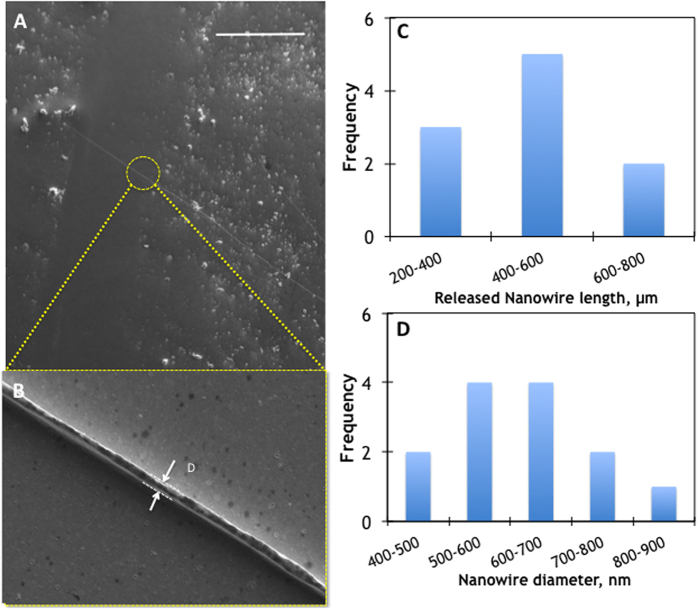
Releasing nanowire core from glass matrix. (**A**) Scanning electron microscopy image of an etched Au nanowire transferred on a planar Au-coated glass substrate (scale bar = 500 μm). (**B**) In this case the measured nanowire diameter is D = 455 nm. (**C,D**) Statistical analysis of variation of the released nanowire core diameter and length in a fibre draw. Measurements taken at stochastically chosen points across several meters of drawn fibre.

From the perspective of large volume nanowire manufacturing, the LAFD method enables production of gigantic aspect ratio nanowires (>10^4^) that can be manipulated as they are enclosed in the glass matrix; this allows deterministic positioning of nanowires over macroscopic distances, with unprecedented freedom. As a term of reference, conventional methods to produce Au nanowires such as electro-deposition in AAO templates typically yield nanowires with lengths not exceeding a few micrometres; furthermore, conventional mechanical positioning of nanowires usually rely on complex and not scalable methods such as Langmuir-Blodgett^50^, blown bubble film^51^, direct printing^52^, or electrochemical deposition 53. In our case, the glass matrix can be easily removed by chemical etching after the nanowire fibres are positioned in desired locations on a planar substrate. Release of the embedded Au nanowires onto a planar Au coated glass substrate is demonstrated in Fig. 5. An appropriate etchant must be chosen depending on the material combination used for the clad and the functional core (we used hydrofluoric acid as it etches the silicate clad without attacking Au and the substrate). As seen in Fig. 5A, after the post-etch wash, portions of the nanowire fibre cores as long as ~500 μm could be successfully released. The measured nanowire diameter shown is of the order of 450 nm (Fig. 5B), and vary by as much as 20% from nanowire end-to-end, reflecting the original diameter variation due to inherent instabilities in the draw giving rise to Rayleigh-Plateu instabilities within the fibre draw^54^,^55^.

## Conclusion

In conclusion, we have introduced a fibre drawing nanomanufacturing technique, called lithography-assisted fibre drawing (LAFD), for the production of ultra-long nanowires in glass claddings. LAFD relies on planar lithography to incorporate heterogeneous thin films into the preform, with pre-patterned features that can be scaled down to nanometres in a single fibre draw. As a proof of principle, we demonstrated Au nanowires embedded into commercial Schott NSF-8 glass fibres, which are structurally and electrically functional over macroscopic lengths, and can be easily handled and manipulated. From a materials perspective, this technique enables the realization of multimaterial nanowire fibres that have constituents with vastly different thermal properties. For instance, besides incorporation of metals as demonstrated here, glasses and semiconductors, whether amorphous or crystalline in phase, could potentially be incorporated with complex patterns within glass matrices; multimaterial nanoscale cores, such as heterogeneous core-shell stacks, could be produced by multiple sequential deposition steps; complex architectures, such as two-dimensional nanowire arrays with arbitrary arrangement, could also be achieved by multiple splicing of the preform. This paves the way to the realization of nanowire heterojunctions and a variety of multimaterial based fiberised devices.

## Experimental Section

### Photolithography

We used S1813 or S1828 positive photoresist, depending on the thickness of material being deposited. A pre-exposure bake, or soft bake, was used before UV exposure to remove the solvent from the resist, typically 1 minute on a 90 °C vacuum hot plate or 30 minutes in a 100 °C convection oven. Any given pattern can be defined using UV exposure through a photomask. The time for UV exposure depends on the resist thickness and optical transparency of the preform material. Post-exposure development is performed right after the UV exposure in a developer solution. The exposed resist was finally developed for about 60 seconds in a metal-ion-free developer (MF319).

### Preform cutting and fusing

The preforms were cut using a standard glass saw and fused back together after photolithographic patterning and deposition. The preforms are typically fused at a temperature 5–10 °C lower than the softening point of the glass. For the N-SF8 preforms we used a furnace at T = 675 °C, after a 10 °C. min^−1^ ramp-up with a 30 minute dwell and a 20 °C.min^−1^ ramp-down to ambient temperature.

### Thermal analysis

In thermogravimetric/differential thermal analysis (Tg/DTA), the material under study and an inert reference are made to undergo identical thermal cycles. Within the DTA signal any temperature difference between sample and reference is recorded. In our experiment, Au was deposited by thermal evaporation into the inner wall of an alumina crucible under high vacuum (P < 10^−5^ mbar). An identical empty crucible was used as a reference. Measurements were conducted in a Perkin Elmer Diamond Tg/DTA instrument.

### Fibre drawing

Fibre drawing was typically carried out in argon purged furnace (flow rate: 1 litre.min^−1^) with a well-defined hot zone at preform temperature of 850 °C. The preform was fed through the hot zone with a feed rate of 1 mm.min^−1^ to gain a bare fibre outer diameter across a range of sizes from 0.5 mm to 50 μm, using a drum speed of 5–15 m.min^−1^. During the fibre draw, the purge gas of Ar completely engulfed the preform, necking region, and vulnerable region of the drawn fibre.

### Fibre cleaving

When cleaving metal cored fibres and in particular metallic nanowire cored fibre, we often found that a portion of the nanowire core is pulled out during the cleave. In this work, best cleaving was achieved using focused ion beam milling at low ion currents to prevent melting of the core.

## Additional Information

**How to cite this article**: Gholipour, B. *et al*. Lithography Assisted Fiber-Drawing Nanomanufacturing. *Sci. Rep.* **6**, 35409; doi: 10.1038/srep35409 (2016).
